# A Modified NK Cell Degranulation Assay Applicable for Routine Evaluation of NK Cell Function

**DOI:** 10.1155/2016/3769590

**Published:** 2016-06-20

**Authors:** Snehal Shabrish, Maya Gupta, Manisha Madkaikar

**Affiliations:** Department of Pediatric Immunology and Leukocyte Biology, National Institute of Immunohaematology (ICMR), 13th Floor, Multistoreyed Building, KEM Campus, Parel, Mumbai 400 012, India

## Abstract

Natural killer (NK) cells play important role in innate immunity against tumors and viral infections. Studies show that lysosome-associated membrane protein-1 (LAMP-1, CD107a) is a marker for degranulation of NK and cytotoxic T cells and its expression is a sensitive marker for the cytotoxic activity determination. The conventional methods of determination of CD107a on NK cells involve use of peripheral blood mononuclear cells (PBMC) or pure NK cells and K562 cells as stimulants. Thus, it requires large volume of blood sample which is usually difficult to obtain in pediatric patients and patients with cytopenia and also requires specialized laboratory for maintaining cell line. We have designed a flow cytometric assay to determine CD107a on NK cells using whole blood, eliminating the need for isolation of PBMC or isolate NK cells. This assay uses phorbol-12-myristate-13-acetate (PMA) and calcium ionophore (Ca^2+^-ionophore) instead of K562 cells for stimulation and thus does not require specialized cell culture laboratory. CD107a expression on NK cells using modified NK cell degranulation assay compared to the conventional assay was significantly elevated (*p* < 0.0001). It was also validated by testing patients diagnosed with familial hemophagocytic lymphohistiocytosis (FHL) with defect in exocytosis. This assay is rapid, cost effective, and reproducible and requires significantly less volume of blood which is important for clinical evaluation of NK cells.

## 1. Introduction

Natural killer (NK) cells are a subset of lymphocytes that play a central role in the innate immune response to tumors and viral infections. These cells kill by a mechanism involving the release of small cytolytic granules containing granzyme B and perforin that induce cell death in the target cell. Although NK cell numbers can be enumerated by immunophenotyping on the flow cytometer, an important limitation in the study of NK cells is the lack of availability of high throughput assays for the detection of the functional activity of NK cells.

During degranulation, cytolytic granules in NK cells are released and the lysosome-associated membrane protein-1 (LAMP-1, CD107a) which is present on cytolytic granules surface is transported to the cell surface and becomes accessible for antibody binding. This allows identification of activated NK cells, making it an attractive biomarker for assessing the integrity of the granule exocytosis mechanism. The assays reported till date for determination of the degranulation marker use peripheral blood mononuclear cells (PBMC) or pure NK cells and K562 cell line as stimulants [1]. PBMC separation or NK cells isolation requires large volume of blood sample which is difficult to obtain especially from pediatric patients below the age of 1 year and patients with cytopenia. Cell separation procedures are time consuming and may also lead to loss of physiological conditions, such as soluble factors like cytokines, growth factors, or chemokines [2]. Using K562 cell line as stimulant requires specialized cell culture facility for maintaining cell line. Thus this assay is not feasible in routine clinical set-up. In 2010, Wheeler et al. reported a degranulation assay using of phytohaemagglutinin (PHA) instead of K562 cells, which made it feasible in clinical set-up [3]. However, in this assay PBMC were used and thus involves cell separation procedure.

For these reasons, we have developed a rapid flow cytometry-based assay for measuring NK cell degranulation using whole blood and phorbol-12-myristate-13-acetate (PMA) and calcium ionophore (Ca^2+^-ionophore) as stimulants. The results were compared with the conventional assay (assay using K562 cells as stimulant) and reproducibility of the assay was evaluated by determining intra-assay and interassay variation.

Hemophagocytic lymphohistiocytosis (HLH) is a disorder of hyperinflammation resulting from impaired clearance of pathogens due to defective NK cell or cytotoxic T cells (CTL). HLH is divided into familial hemophagocytic lymphohistiocytosis (FHL) resulting from inherited defect in the granule mediated cytotoxicity of cytotoxic cells (NK cells and CTL) and acquired HLH which is caused due to overwhelming trigger of viral infections, malignancies, or rheumatic diseases. FHL can be further subclassified into FHL2 caused due to perforin deficiency (FHL-2; MIM 603553) and genetic defects affecting proteins involved in transport, membrane fusion, or exocytosis of perforin containing lytic granules such as Munc 13-4 (FHL-3; MIM 608898), syntaxin 11 (FHL-4; MIM 603552), and syntaxin binding protein 2 (Munc 18-2) (FHL-5; MIM 613101) [4]. Griscelli syndrome type 2 (GS2) and Chediak-Higashi syndrome (CHS), caused by mutations in* RAB27A *and* LYST*, respectively, are also associated with development of HLH. These patients display impaired lymphocyte cytotoxicity and in addition manifest partial albinism [5]. Diagnosis of FHL is important as these patients need hematopoietic stem cell transplantation (HSCT) for long term survival. In literature, the NK cell degranulation assay has been shown to be useful in diagnosis of familial hemophagocytic lymphohistiocytosis (FHL) associated with defect in the granule release mechanism (FHL3–5, CHS, GS2) [4, 6]. However in FHL2 patients, perforin protein is defective whereas the granule release mechanism is not affected and hence this assay is expected to be normal in these patients. Hence, to validate the usefulness of this assay in a clinical laboratory, it was performed in different groups of HLH patients and the outcome was correlated with the diagnosis.

## 2. Materials and Methods

### 2.1. Blood Samples

#### 2.1.1. Normal Samples

Fresh heparinized peripheral blood samples were collected from 30 healthy donors 20 to 55 years of age, including both males and females.

#### 2.1.2. HLH Patient Samples

HLH diagnosis was carried out based of diagnostic criteria proposed by Histiocyte society [7]. Further diagnosis of FHL2 (perforin deficiency) was carried out by flow cytometry and that of FHL3 (Munc 13-4 deficiency) and FHL4 (syntaxin 11 deficiency) by Western blotting for respective proteins and also molecular characterization of the respective genes. Chediak Higashi syndrome (CHS) and Griscelli syndrome 2 diagnosis was based on clinical manifestations and microscopic examination of hair mount and peripheral blood smear.

For validation of the proposed assay total, 19 FHL [1 FHL2, 7 FHL3, 1 FHL4, 2 Chediak Higashi Syndrome (CHS), and 1 Griscelli syndrome type 2] and 8 acquired HLH patients were evaluated.

#### 2.1.3. Non-HLH Patient Samples

To determine the specificity of the assay, 18 patients with infections (namely, 4 viral, 11 bacterial, and 3 fungal) and receiving treatment for the same were also evaluated using this assay. This group consisted of patients of age ranging from 7 days to 60 years, both male and female.

Each subject gave informed consent for participation in the study after the nature and possible consequences of the studies had been fully explained.

### 2.2. Stimulants

#### 2.2.1. k562 Cell Line

K562 cells (a human erythroleukemia cell line) were cultured in RPMI 1640 media (GIBCO, Life Technologies) supplemented with 10% (v/v) fetal bovine serum (FBS, Sigma), L-glutamine at 37°C under 5% (v/v) CO_2_.

#### 2.2.2. Phorbol-12-Myristate-13-Acetate (PMA)

1.5 *μ*L of 1 mg/mL PMA (Sigma Chem. Co., St. Louis, MO) was added to 2.5 mL of incomplete RPMI 1640 media to obtain concentration of 0.6 *μ*g/mL. Serial dilution with incomplete media was carried out to obtain final concentration of 0.15 *μ*g/mL.

#### 2.2.3. Ca^2+^ Ionophore

4.5 *μ*L of 1 mg/mL Ca^2+^ ionophore (Ionomycin, Sigma Chem. Co., St. Louis, MO) was added to 1.5 mL of incomplete RPMI 1640 media to obtain final concentration of 3 *μ*g/mL.

### 2.3. Sample Preparation

Absolute white blood cells (WBC) count and absolute lymphocyte count (ALC) was determined using Sysmex XS-800i cell counter.

### 2.4. Detection of CD107a Expression by Flow Cytometry

For each sample 3 tubes were processed, namely, unstimulated, K562 stimulated, and PMA + Ca^2+^-ionophore stimulated. 100 *μ*L of whole blood sample (ALC ≈ 2 × 10^5^ cells/mL) and 5 *μ*L FITC conjugated anti-CD107a (BD Biosciences) were added to each tube.

#### 2.4.1. K562 Cells Stimulation

200 *μ*L of 2 × 10^6^/mL K562 cells was added to the K562 stimulated tubes.

#### 2.4.2. PMA + Ca^2+^-Ionophore Stimulation

100 *μ*L of PMA (0.15 *μ*g/mL) and 100 *μ*L Ca^2+^ ionophore (3 *μ*g/mL) were added to the PMA + Ca^2+^-ionophore stimulated tubes and the final volume was adjusted to 500 *μ*L with incomplete RPMI 1640 media.

All the tubes were incubated for 2 h at 37°C under 5% (v/v) CO_2_. Unstimulated samples were incubated without stimulants to detect spontaneous degranulation.

Thereafter, samples were washed with PBS and stained with anti-CD56-PE (NCAM16.2) and anti-CD3APC (BD Bioscience, San Jose, CA), followed by erythrocyte lysis of blood samples using FACS Lysing solution. The flow cytometric analysis was performed on FACSAria using DIVA software (Becton Dickinson, San Jose, CA, USA).

*Flow Cytometry Analysis*. Lymphocytes were gated by forward/side scatter and NK cells based on CD56^+^CD3^−^. The cut-off was set on unstimulated tubes and the increased CD107a expression in the same sample after stimulation was noted.

The increase in CD107a expression using K562 as stimulant and PMA-Ca^2+^ ionophore as stimulants was compared using statistical tests.

*Statistical Analysis*. Data is presented as median ± standard deviation. Two-tailed *t*-test was used to compare assays and also normal controls and FHL patients. The results were reported to be statistically significant if the *P* value was < 0.05.

## 3. Results and Discussion

### 3.1. Comparing Whole Blood and PBMC Samples for NK Cell Degranulation Assay 

In our study, the NK cell degranulation assay results obtained using whole blood (26 ± 4%) and PBMC (25 ± 4%) were comparable. Till date only one article of comprehensive analysis of NK cell function in whole blood samples has been published [5]. In this report, CD107a surface expression was evaluated by stimulating NK cells with K562 cells using whole blood sample and no significant difference in the results was observed.

However, this assay requires K562 cells and thus specialized laboratory with well established cell culture facilities and experienced personnel is essential for maintaining the cell line. Cell cultures are susceptible to bacterial contamination, which affects the assay outcome. It has also been reported that variation in this assay may be observed because of the difference in the source and passage of K562 stimulator cells [1]. This all adds to the cost in clinical set-up and also performing the assay on urgent pediatric samples is not always feasible.

Thus, considering these difficulties, here we report NK cell degranulation assay using whole blood sample instead of pure cells and PMA + Ca^2+^-ionophore as NK cell stimulants instead of conventional stimulant K562 cell line.

### 3.2. Comparing K652 Cell Line and PMA + Ca^2+^-Ionophore as NK Cell Degranulation Stimulants

**Figure 1:**
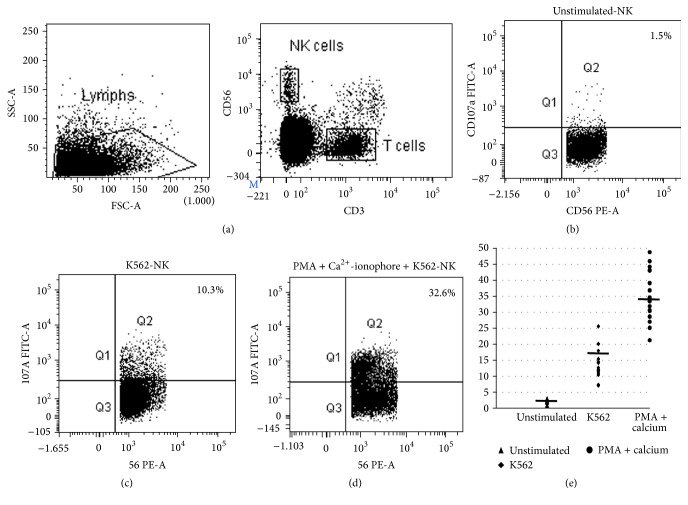
Comparison of conventional degranulation assay with modified assay. Whole blood was incubated with fluorescein isothiocyanate-conjugated antiCD107a antibody alone or with PMA + Ca^2+^-ionophore or K562 cells for 2 h (as three separate samples). Samples were analyzed by flow cytometry, gating on lymphocytes by forward/side scatter. CD107a expression was analyzed on natural killer (NK) cells (CD56^+^CD3^−^), (a) and the increase in % CD107a^+^ NK cells between unstimulated and stimulated samples was calculated. Results were considered valid if the healthy control sample processed along with the patient sample gave normal results. Patient results were considered abnormal if the increase was <10% on stimulation. Flow cytometry figures represent the percent NK cells that express CD107a following no stimulation (b) and stimulation with K562 cells (c) or with PMA + Ca^2+^-ionophore (d) in a single representative subject. The dot plot represents the percent NK cells that express CD107a following no stimulation and stimulation with K562 cells or PMA + Ca^2+^-ionophore for all subjects tested in this study (e).

K562 cell line is MHC I devoid and hence it acts as target cells for NK cells. Whereas PMA is a substitute for diacylglycerol (DAG), one of the adaptor proteins required for the activation of protein kinase C [8, 9] and Ca^2+^-ionophore increases intracellular calcium levels [10]. Therefore, the combination of PMA + Ca^2+^-ionophore facilitates the activation of protein kinase C and an influx of intracellular calcium which are the necessary signaling events for degranulation [8, 11]. On comparing assays using these stimulants, it was observed that the difference in upregulation of CD107a expression on NK cells by stimulation with PMA + Ca^2+^-ionophore (34.05 ± 8.44) was significantly higher than that with K562 cells (13.7 ± 4.18) (*p* < 0.0001) (Figure 1). Since ΔCD107a is higher by using PMA + Ca^2+^-ionophore, it allows the clear distinction of patients with normal and abnormal granule release mechanism especially in case of borderline degranulation as reported in few patients with late-onset FHL4 and FHL5 [1]. Previously, Alter et al. (2004) had demonstrated upregulation of CD107a expression on NK cells stimulated with PMA/Ionomycin (average frequency 21.5%) [12]. In this study, PBMC were used, the concentration of PMA and Ionomycin used were 2.5 *μ*g/mL and 0.5 *μ*g/mL, respectively, and the incubation time was 6 h in presence of monensin (to avoid internalization of CD107a). However in the assay proposed in our study, whole blood was used and in spite of incubating the samples for only 2 h without monensin; the average frequency observed was 35.6 ± 6.95% (*n* = 30; range 21.3–48.8%), which is much higher than reported. The interassay and intra-assay coefficient variation for this assay were 3.66% and 5.17%, respectively, thus demonstrating reproducibility of the assay.

#### 3.2.1. Validation of Modified Degranulation Assay for Clinical Usefulness

**Figure 2:**
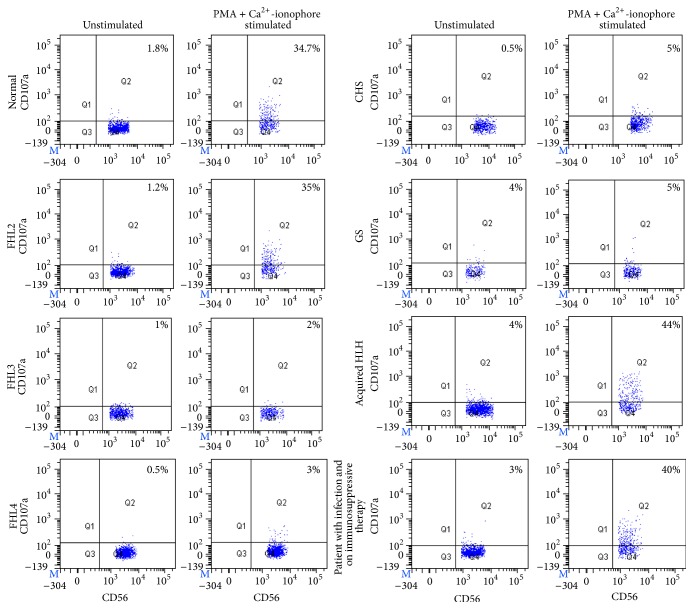
Comparison of CD107a expression on PMA + Ca^2+^ ionophore stimulated NK cells. Degranulation assay results are shown from a normal healthy control and patients with FHL2 (mutations in* PRF1* encoding perforin), FHL3 (mutations in UNC13D encoding Munc13-4), FHL4 (mutations in STX11 encoding syntaxin-11), Chediak Higashi syndrome (CHS, mutations in* LYST*), Griscelli syndrome type 2 (GS2, mutations in* RAB27a*), acquired HLH, and non-HLH patients with infections and receiving treatment for the same. The percentage of CD107a^+^ NK cells is indicated on each plot. In FHL2 patients, acquired HLH patients, non-HLH patients with infection, and healthy controls the CD107a expression on NK cells increased significantly after stimulation. However, in FHL3, FHL4, CHS, and GS2 patients it did not increase, indicating defective granule release mechanism.

Hemophagocytic lymphohistiocytosis (HLH) is a disorder of immune dysregulation and is classified into familial HLH (FHL characterized by defect in granule exocytosis) and acquired HLH. Specifically, patients with FHL3, FHL4, FHL5, Chediak Higashi syndrome, and Griscelli syndrome are known to have defective granule exocytosis and hence have low expression of CD107a expression on stimulated NK cells. FHL2 patients have defect in perforin expression with normal granule release mechanism whereas acquired HLH patients have no defect in perforin expression and granule release mechanism [4]. Hence, to validate this assay, 19 HLH patients, including 1 FHL2 (*PRF1* gene defect), 7 FHL3 (*UNC13D* gene defect), 1 FHL4 (*STX11* gene defect), 2 CHS, 1 GS2, and 8 acquired HLH patients were analyzed. Amongst these, as expected FHL3, FHL4, GS2, and CHS patients had low expression of ΔCD107a on stimulated NK cells (0.2–4.7%) compared to FHL2 patients (41.8%), acquired HLH patients (21.6–48%), and healthy controls (21.3–48.8%) (*p* < 0.0001) (Figure 2). This assay was not tested in FHL5 patients.

To determine the specificity of this assay, it was carried out in 11 patients with bacterial infection, 4 with viral infections, and 3 with fungal infections; 8 of these patients were receiving treatment for the same. All these patients had expression of CD107a on NK cells in normal range (20–46%; normal range 21.3–48.8%), indicating the results of this assay are not influenced by the infection or therapy.

## 4. Concluding Remarks

To conclude, CD107a is a reliable marker of NK cell cytotoxicity and helps in comprehensive evaluation of NK cell function. Since, in the described assay, whole blood is used and no cell line is used, it requires reduced blood volume, is cost-effective, is less time consuming, and avoids necessity for specialized laboratory for cell culture, which makes it applicable in routine clinical set-up. Along with the existing perforin assay for identifying FHL2 patients, this assay aids in distinguishing FHL patients with defective granule release mechanism (namely, FHL3, FHL4, GS, and CHS) from acquired HLH patients which is essential in their management.
